# Predicting response of hepatoblastoma primary lesions to neoadjuvant chemotherapy through contrast-enhanced computed tomography radiomics

**DOI:** 10.1007/s00432-024-05746-x

**Published:** 2024-04-30

**Authors:** Yanlin Yang, Haoru Wang, Jiajun Si, Li Zhang, Hao Ding, Fang Wang, Ling He, Xin Chen

**Affiliations:** 1 Department of Radiology, Children’s Hospital of Chongqing Medical University, National Clinical Research Center for Child Health and Disorders, Ministry of Education Key Laboratory of Child Development and Disorders, Chongqing Key Laboratory of Child Neurodevelopment and Cognitive Disorders, Chongqing, China; 2 Department of Research and Development, Shanghai United Imaging Intelligence Co., Ltd, Shanghai, China

**Keywords:** Children, Computed tomography, Hepatoblastoma, Radiomics, Neoadjuvant chemotherapy

## Abstract

**Objective:**

To investigate the clinical value of contrast-enhanced computed tomography (CECT) radiomics for predicting the response of primary lesions to neoadjuvant chemotherapy in hepatoblastoma.

**Methods:**

Clinical and CECT imaging data were retrospectively collected from 116 children with hepatoblastoma who received neoadjuvant chemotherapy. Tumor response was assessed according to the Response Evaluation Criteria in Solid Tumors (RECIST). Subsequently, they were randomly stratified into a training cohort and a test cohort in a 7:3 ratio. The clinical model was constructed using univariate and multivariate logistic regression, while the radiomics model was developed based on selected radiomics features employing the support vector machine algorithm. The combined clinical–radiomics model incorporated both clinical and radiomics features.

**Results:**

The area under the curve (AUC) for the clinical, radiomics, and combined models was 0.704 (95% CI: 0.563–0.845), 0.830 (95% CI: 0.704–0.959), and 0.874 (95% CI: 0.768–0.981) in the training cohort, respectively. In the validation cohort, the combined model achieved the highest mean AUC of 0.830 (95% CI 0.616–0.999), with a sensitivity, specificity, accuracy, precision, and f1 score of 72.0%, 81.1%, 78.5%, 57.2%, and 63.5%, respectively.

**Conclusion:**

CECT radiomics has the potential to predict primary lesion response to neoadjuvant chemotherapy in hepatoblastoma.

**Supplementary Information:**

The online version contains supplementary material available at 10.1007/s00432-024-05746-x.

## Introduction

Hepatoblastoma (HB) is the most common liver malignancy in children, with an annual incidence of 1.5 cases per million children (Rougemont et al. 2012). However, only 30% of tumors are directly amenable to surgical resection at initial diagnosis (Meyers et al. 2017). According to the International Childhood Liver Tumors Strategy Group (SIOPEL) program, neoadjuvant chemotherapy should be administered in all cases irrespective of the pretreatment extent of disease (PRETEXT) staging of HB. The SIOPEL has found that a platinum-based chemotherapy regimen can lead to improved satisfaction with tumor cytoreduction. This regimen can also result in more complete tumor resection and a reduction in the incidence of postoperative adverse events and mortality (Katzenstein et al. 2019; Zsiros et al. 2013; Hiyama et al. 2020). Therefore, neoadjuvant chemotherapy is a crucial component of HB treatment, as it significantly increases the rate of surgical resection to 74–95%, reduces metastasis and recurrence, and ultimately improves patient outcomes (Marin et al. 2019).

However, due to the heterogeneity of HB, tumor response to neoadjuvant chemotherapy varies. Children with pure fetal histology HB treated with complete surgical resection and minimal adjuvant therapy have been shown to have excellent outcomes when compared with other patients (Malogolowkin et al. 2011). Katzenstein et al. (2017) found that only 14 out of 30 patients with HB responded to chemotherapy. According to a study conducted by Venkatramani et al. (2015), among the 20 patients with PRETEXT types III and IV, most achieved surgical readiness after 2 cycles of chemotherapy. However, for four children, surgical resection was not possible even after 4 cycles of chemotherapy, and they had to undergo liver transplantation. For these drug-resistant patients, targeted drugs and immunotherapeutic approaches should be an additional option for preoperative treatment (Hiyama et al. 2016). Therefore, predicting the response of HB to neoadjuvant chemotherapy helps clinicians to modulate the intensity of treatment and select the appropriate treatment regimen, ultimately predicting the prognosis of patients.

Some studies indicate that there are differences in the transcriptomic signature and epigenetic machinery of HB with varied treatment responses. These findings contribute to a deeper understanding of HB development and therapeutic responses, offering potential predictive biomarkers for personalized therapy (Song et al. 2022; Clavería-Cabello et al. 2023). However, the development of a simple and practical predictive model for chemotherapy response in HB is still a challenge. In recent years, radiomics can serve as a noninvasive tool to offer a comprehensive picture of tumor heterogeneity (Wang et al. 2023a, b). Radiomics features are widely used to predict the response to neoadjuvant chemotherapy in various types of solid tumors in both adults and children, including neuroblastoma and nephroblastoma. Studies have shown promising results (Xu et al. 2021; Wang et al. 2021; Choudhery et al. 2022; Wang et al. 2023a, b; Sharaby et al. 2023). However, there are limited reports on the use of contrast-enhanced computed tomography (CECT) radiomics to predict the response of HB to neoadjuvant chemotherapy.

Therefore, the objective of this study was to investigate the clinical value of CECT radiomics for predicting the response of primary lesions to neoadjuvant chemotherapy in HB.

## Materials and methods

### Patients

**Fig. 1:**
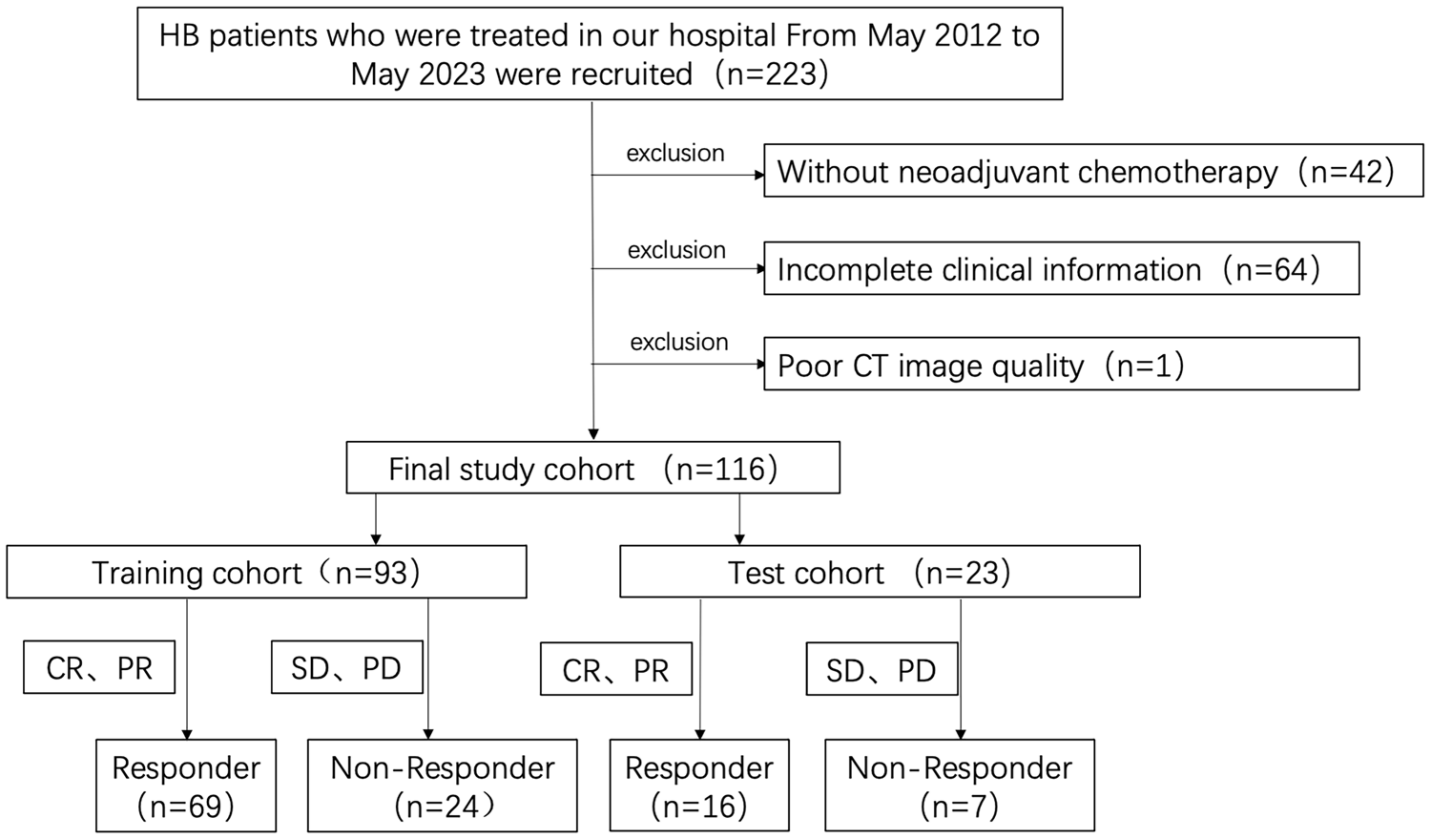
Study flowchart of the enrolled patients. CR: complete remission; PR: partial remission; SD: stable disease; PD: progression disease

This was a retrospective study that was approved by the Ethics Committee of the Children’s Hospital of Chongqing Medical University (File No. 2023-472), and patients’ informed consent was waived. Patients with HB admitted to our hospital from May 2012 to May 2023 were retrospectively collected. Patients who met the following criteria were eligible for enrollment: (1) patients received neoadjuvant chemotherapy; (2) patients underwent CECT before neoadjuvant chemotherapy; (3) patients underwent CECT or MRI after treatment to assess the response of the primary lesion; and (4) two to four cycles of neoadjuvant chemotherapy between the two exams; the exclusion criteria were as follows: (1) incomplete clinical data before and after neoadjuvant chemotherapy and (2) poor image quality (Fig. 1).

The effectiveness of chemotherapy was assessed based on the Response Evaluation Criteria in Solid Tumors (RECIST), and patients were categorized into either a responder group or a non-responder group, according to the degree of tumor shrinkage (Therasse et al. 2000). Subsequently, the participants were divided into a training cohort and a test cohort in a randomized 7:3 ratio.

### CECT scanning protocol

The patient underwent a GE Lightspeed 64/Philips Brilliance 256 spiral CT scan at the time of initial diagnosis with the following parameters—tube voltage 80–100 kV, tube current 150–200 mA s, acquisition slice thickness 5.0 mm, pitch 1.1 mm, collimation 0.6 mm, and reconstruction slice thickness 1.25 mm. CT scanning was performed with iodixanol, iodine concentration of 320 mg/l, dose of 1.5–2.0 ml/kg and flow rate of 0.5–3.5 ml/s. Arterial and venous phase scans were performed 20–28 s and 55–66 s after contrast injection, respectively.

### Clinical model construction

The patients in the responder group and non-responder group were compared based on age, gender, distant metastases, alpha-fetoprotein at initial diagnosis, PRETEXT, and PRETEXT annotation factor (VPEFR). VPEFR includes V hepatic vein/inferior vena cava, P portal vein, F multifocal, R tumor rupture, and E extrahepatic tumor extension. In addition, a combined factor of VPEFR was identified as positive (VPEFR+) if one of the V, P, E, F, or R factors, as described by CHIC-HS, were present. Clinical characteristics were analyzed using univariate logistic regression analysis in the training cohort. Only those with a *P* value less than 0.05 were included in the multivariate logistic regression.

### Region of interest segmentation and feature extraction

Tumor segmentation and radiomics feature extraction were carried out using the uAI Research Portal software (version 20231115). To reduce differences in image specifications, window width and window position normalization, and image resampling (1 × 1 × 1 mm^3^) were performed on the images. The region of interest (ROI) was manually and blindly delineated by a radiologist with 3 years of experience along the contours of the lesion on the portal venous phase images. All delineated ROIs were reviewed and corrected by another radiologist with 15 years of experience. To evaluate the reproducibility of the radiomics features, approximately 1/3 of the cases were randomly selected from the training cohort and delineated again. A total of 2264 radiomics features were extracted from each ROI, including 18 first-order features; 14 shape-based features; 21 Gray-Level Co-occurrence Matrix (GLCM) features; 16 Gray-Level Size Zone Matrix (GLSZM) features; 16 Gray-Level Run-Length Matrix (GLRLM) features; 5 Neighborhood Gray-Tone Differences Matrix (NGTDM) features; 14 Gray-Level Dependence Matrix (GLDM) features; 2160 higher order statistical features from raw images that had been processed with 24 filters (box-mean, additive Gaussian noise, binomial blur, curvature flow, box-sigma, normalization, Laplace sharpening, discrete Gaussian, mean, speckle noise, recursive Gaussian, shot noise, LoG (sigma: 0. 5, 1, 1.5, 2), and wavelet (LLL, LLH, LHL, LHH, HHL, HLH, HHL, HHH)).

### Selection of radiomics features

All features were normalized using *z*-score standardization to eliminate the dimensional effects of different features. For radiomics features extracted from twice delineations, intraclass correlation coefficient (ICC) analysis was performed, and radiomics features with an ICC greater than 0.75 were considered reproducible radiomics features and selected for further analysis. Finally, the best radiomics features were selected by the variance thresholding method (threshold = 0.75), the Max-Relevance and Min-Redundancy (MRMR) (feature retention = 15), and the Least Absolute Shrinkage and Selection Operator (LASSO).The hyperparameters of the LASSO were determined by five-fold cross-validation.

### Radiomics and clinical–radiomics model building

To prevent overfitting in this small sample size study, we constructed a radiomics model using the support vector machine (SVM) algorithm based on the final selected radiomics features, and similarly, the optimal clinical and radiomics features were selected to build a clinical–radiomics model, and the C, kernel, gamma, and threshold of the SVM algorithm were 1.0, rbf, 0.01, and 0.5, respectively.

### Model performance evaluation

**Fig. 2:**
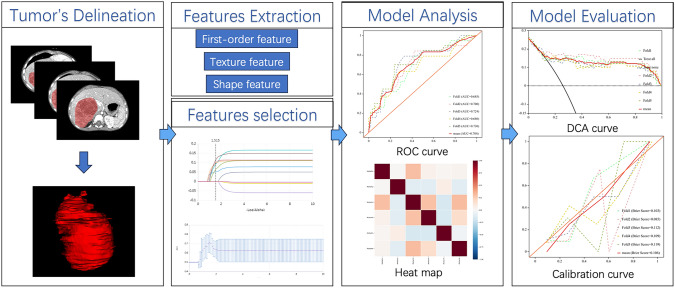
Flowchart showing the process for the development of radiomics

To avoid sample bias in grouping, fivefold cross-validation was used to validate the model. Receiver operating characteristic (ROC) curves were plotted to evaluate the predictive performance of each model in the training and validation cohorts. The area under the ROC curve (AUC), 95% confidence interval (CI), sensitivity, specificity, accuracy, and f1 score have been calculated. The calibration curves of each model in both the training and validation cohorts were plotted. In addition, decision curve analysis (DCA) was utilized to evaluate the clinical validity of the models by quantifying the net gain at various threshold probabilities. AUC values were compared between models by the Delong test (Fig. 2).

### Statistical analysis

IBM SPSS Statistics software (version 25.0) and uAI Research Portal software (version 20231115) were used for statistical analysis. The abnormally distributed quantitative variables were compared using a Wilcoxon test. The qualitative variables were tested using a Chi-square test or Fisher’s test. A statistically significant difference was considered when the two-tail *P* value was less than 0.05.

## Results

### Clinical features

The study enrolled a total of 116 patients diagnosed with HB, including 76 males and 40 females, with a mean age of 23.14 months (range: 3–139 months). There were 85 patients in the responder group, with 57 males and 28 females, and the mean age was 19.56 months (range: 3.0–126.0 months). The non-responder group consisted of 31 patients, with 19 males and 12 females, and the mean age was 32.71 months (range: 3–139 months). The training cohort had a total of 93 patients, of which 69 were responders and 24 were non-responders. The test cohort had a total of 23 patients, including 16 responders and 7 non-responders (Table 1).

**Table 1:** Patient clinical and pathological characteristics

Clinical features	Training cohort (*n* = 93)			Test cohort (*n* = 23)		
	Responder	Non-responder	*P* value	Responder	Non-responder	*P* value
Gander			0.273			0.621
Male	46 (66.7%)	13 (54.2%)		11 (68.8%)	6 (85.7%)	
Female	23 (33.3%)	11 (45.8%)		5 (31.3%)	1 (14.3%)	
Age (months)	15 (8, 23)	24 (10, 46)	0.036	17.5 (7, 33.25)	11 (6, 34)	0.599
PRETEXT			0.006			0.772
I	2	0		0	1	
II	4	0		3	0	
III	43	10		7	4	
IV	20	14		6	2	
AFP (×10^−5^ μg/L)	4.1 (1.26, 8.1)	7.6 (5.9, 13.0)	0.586	8.1 (2.1, 16.4)	4.5 (2.4, 13.8)	0.423
VPEFR+	12 (17.4%)	11 (45.8%)	0.005	3 (18.8%)	1 (14.3%)	1
M+	7 (10.1%)	8 (33.3%)	0.02	4 (25.0%)	2 (28.6%)	1

PRETEXT = Pretreatment Extent of Disease. AFP in newborn drawn postoperatively; VPEFR+: Vmacrovascular involvement of all hepatic veins (V) or portal bifurcation (P), contiguous extrahepatic tumor (E), multifocal tumor (F), and spontaneous rupture (R); M+: distant metastasis

### Clinical model performance

The effects of age, PRETEXT, VPEFR+, and M+ on chemotherapy response were statistically significant in univariate analysis, with *P* values of 0.009, 0.008, 0.007, and 0.011, respectively. Incorporating clinical characteristics with correlations into a multivariate logistic analysis revealed that age had a significant effect on chemotherapy response. It was also used as an independent predictor for clinical modeling. According to the multivariate analysis (Table 2), age was identified as an independent risk factor for predicting response to chemotherapy. This information was used to build the clinical model.

**Table 2:** Univariate and multivariate analyses results of clinical features

Clinical features	Univariate		Multivariate	
	OR (95% CI)	*P* value	OR (95% CI)	*P* value
Age	1.154 (0.720–1.791)	0.009	1.891 (1.110–3.481)	0.027
Pretext	2.200 (1.264–4.067)	0.008	2.010 (1.046–4.163)	0.057
AFP	1.154 (0.72–1.791)	0.528		
VPEFR+	1.828 (1.178–2.863)	0.007	1.245 (0.718–2.108)	0.419
M+	1.734 (1.132–2.687)	0.011	1.226 (0.726–2.065)	0.440

### Radiomics feature selection

From the 2264 radiomics features extracted, 1592 features were obtained after retaining the features with ICC greater than 0.75. Due to the *Z*-score normalization of the features, 1592 features remained after further filtering by the variance threshold method. The training cohort has been partitioned into five distinct training set and validation cohorts utilizing a fivefold cross-validation technique. This method involves dividing the training cohort into five equally sized subsets, four of which are utilized as the training set, while the remaining one is used as the validation cohort. In addition, a tenfold within-group cross-validation strategy has been employed to enhance the model’s stability. The features of each fold were screened using the mRMR and LASSO algorithms. The results of the five feature selections were then combined using voting ensembles (Supplementary Table S1). Features that appeared more than twice were considered stable, resulting in the identification of six stable features. The six features, namely, “wavelet-LLL_glrlm_RunEntrop”, “wavelet.LHL_glszm_LAHGLE”, “specklenoise_firstorder_Kurtosis”, “boxsigmaimage_ngtdm_Coarseness”, “normalize_firstorder_Entropy”, and “original_shape_Sphericity”.

### Performance comparison of different models

The mean AUC for the clinical, radiomics, and combined models was 0.704 (95% CI: 0.563–0.845), 0.830 (95% CI: 0.704–0.959), and 0.874 (95% CI: 0.768–0.981) in the training cohort, respectively. In the validation cohort, the combined model achieved the highest mean AUC of 0.830 (95% CI 0.616–0.999), with a sensitivity, specificity, accuracy, precision, and f1 score of 72.0%, 81.1%, 78.5%, 57.2%, and 63.5%, respectively (Table 3).

**Table 3:** Performance of clinical, radiomics, and combined models in the training and validation cohorts

Model	AUC	Sensitivity	Specificity	Accuracy	Precision	f1 score
Radiomics						
Training cohort	0.830 (0.704–0.959)	0.751	0.783	0.774	0.546	0.632
Validation cohort	0.802 (0.543–0.995)	0.680	0.681	0.678	0.416	0.513
Clinical						
Training cohort	0.704 (0.563–0.845)	0.532	0.713	0.666	0.409	0.445
Validation cohort	0.690 (0.381–0.982)	0.530	0.710	0.667	0.402	0.444
Combined						
Training cohort	0.874 (0.768–0.981)	0.761	0.873	0.844	0.676	0.716
Validation cohort	0.830 (0.616–0.999)	0.720	0.811	0.785	0.572	0.635
Test cohort	0.741 (0.499–0.983)	0.571	0.938	0.826	0.800	0.666

**Fig. 3:**
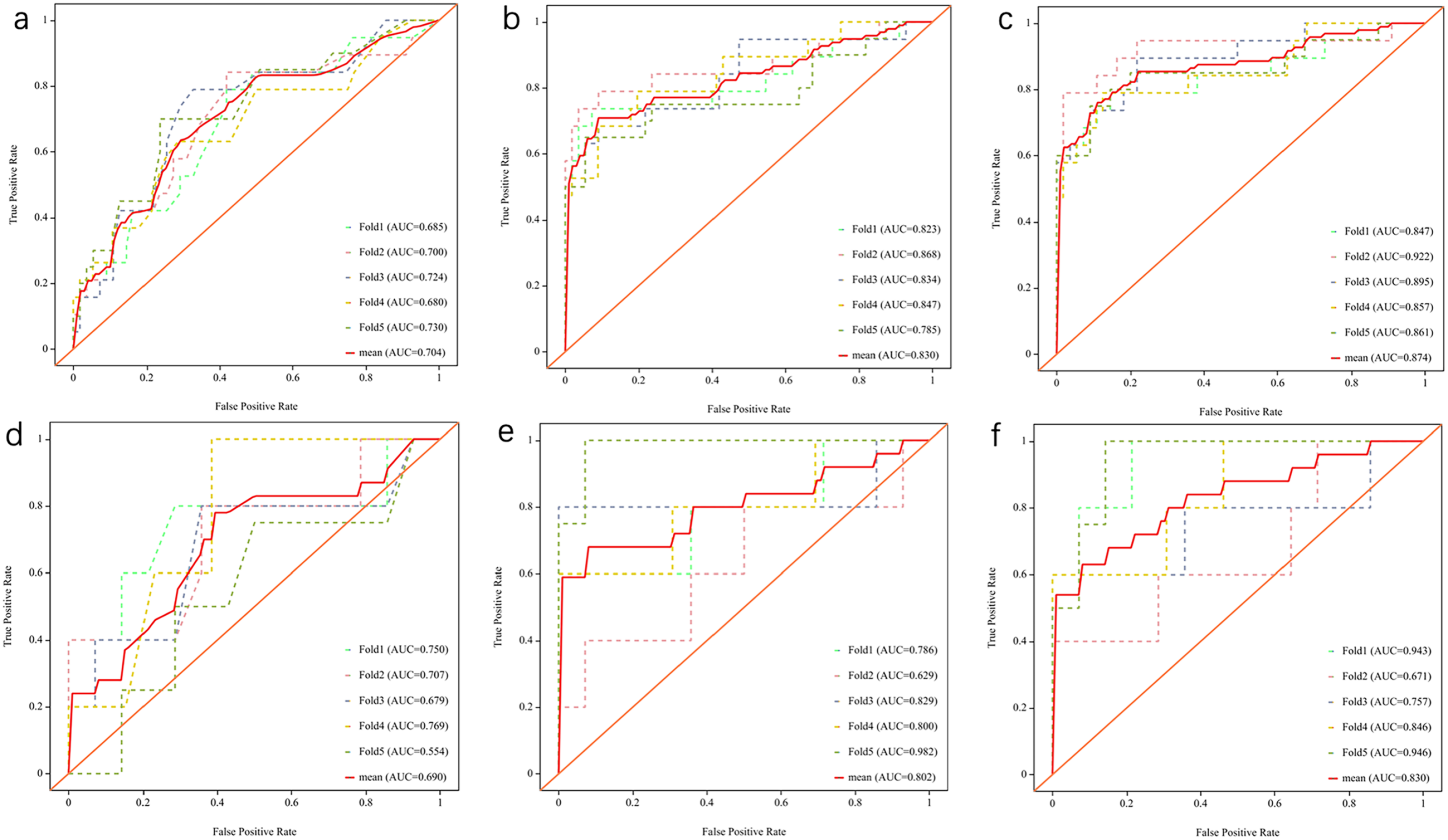
ROC curves of clinical models, radiomics models, and combined models in the training (**a**–**c**) and validation (**d**–**f**) cohorts

According to the Delong test (Supplementary Table S2), there was no statistically significant difference between the diagnostic efficacy of the radiomics model and the clinical model in the training cohorts, but the difference between the diagnostic efficacy of the combined model and the clinical model was statistically significant (*P* = 0.009), which also suggests that the radiomics features have an advantage in improving clinical diagnostic performance (Fig. 3).

**Fig. 4:**
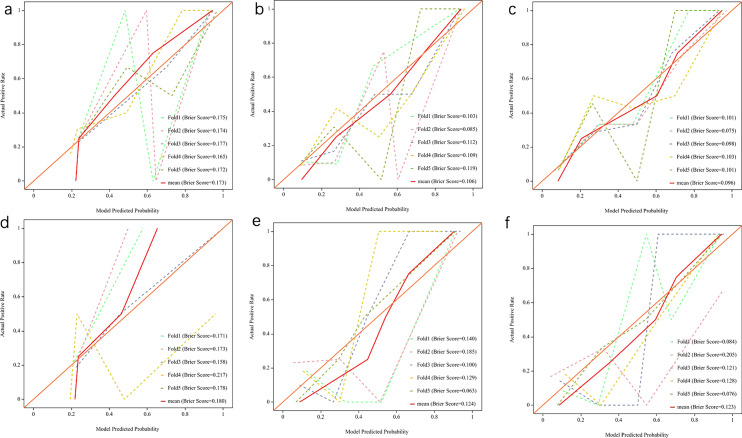
Calibration curves of clinical models, radiomics models, and combined models in the training (**a**–**c**) and validation (**d**–**f**) cohorts

Due to the small sample size, there was no significant difference in the diagnostic efficacy of the three models in our test cohorts. The calibration curves indicated a high level of agreement between predicted and actual probabilities (Fig. 4).

**Fig. 5:**
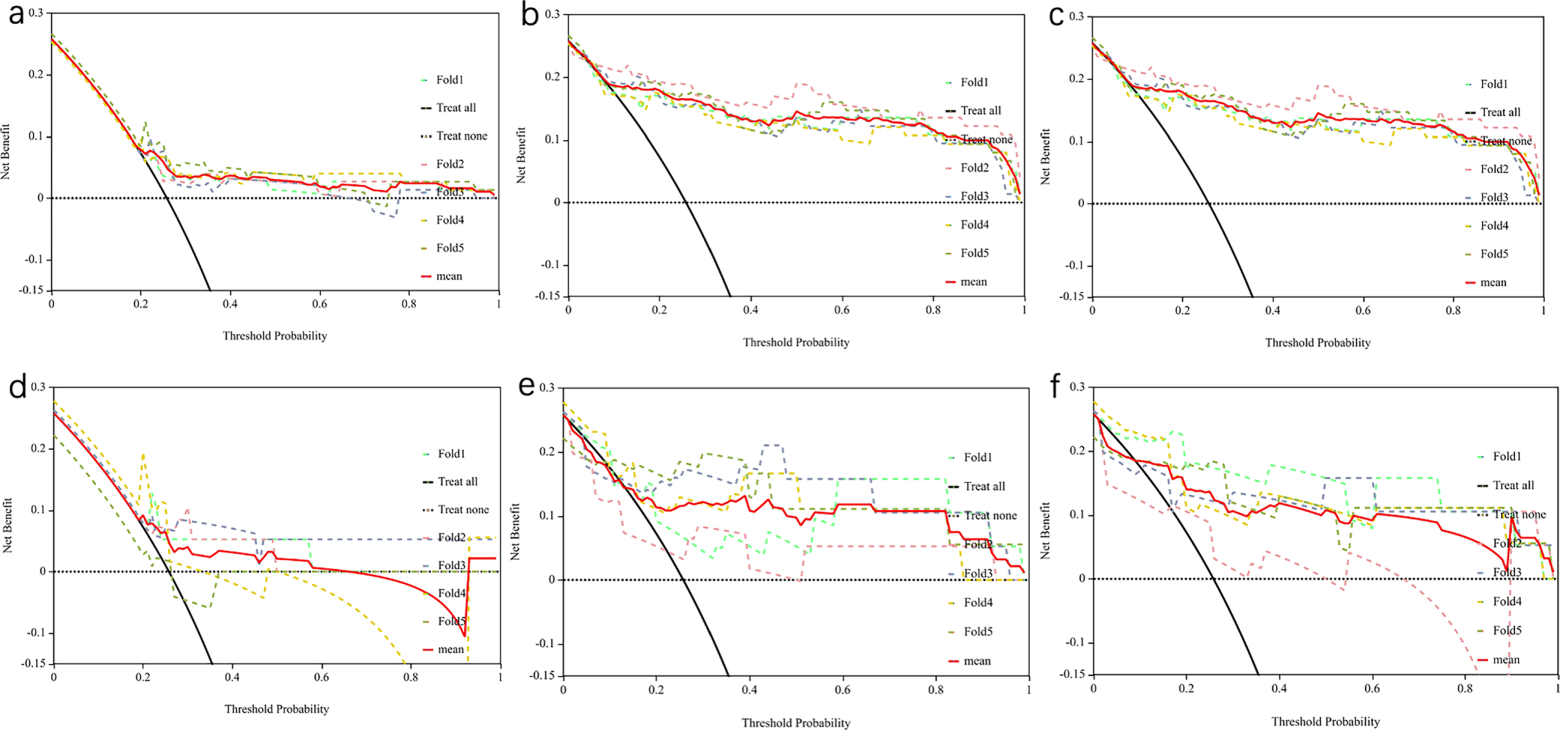
Decision curves of clinical models, radiomics models, and combined models in the training (**a**–**c**) and validation (**d**–**f**) cohorts

According to the decision curve analysis, it appears that the combined model may offer greater clinical utility when compared to the use of radiomics features or clinical features alone (Fig. 5).

## Discussion

The current consensus on chemotherapy for HB is to attempt early resection after two cycles of chemotherapy when the tumor is amenable to surgery. After four cycles, the tumor remains inoperable and liver transplantation is recommended rather than further chemotherapy (Meyers et al. 2012). More chemotherapy instead leads to drug resistance in patients (Venkatramani et al. 2015). Therefore, this study mainly included patients with HB who were evaluated after 2–4 cycles of chemotherapy. Current clinical risk stratification still depends on imaging and histologic features at diagnosis and the molecular marker serum AFP (Pritchard et al. 2000). Clinical factors associated with HB include patient age, pretext staging, PRETEXT annotation factors, and alpha-fetoprotein (Wei et al. 2022). Haeberle et al. (2020) studied and analyzed the clinical data of 1605 children with HB and showed that the older the patient, the higher the risk of extrahepatic metastasis, AFP < 100 ng/ml, and tumor rupture, and the worse the prognosis. In addition, several previous trials have demonstrated that the PRETEXT group is a strong predictor of overall survival in children with HB (Perilongo et al. 2004). In the present study, it was observed that there were no statistically significant differences between the two groups in terms of gender, PRETEXT staging, PRETEXT annotation factors, and extrahepatic abdominal organ involvement. However, a significant difference was found in the age at onset, with the responder group generally being younger. This suggests that a younger age at onset may be associated with better remission after induction chemotherapy. In addition to building a clinical model, we also investigated a radiomics model for predicting response to neoadjuvant chemotherapy based on CECT. The results suggest that radiomics may be useful in predicting responder in HB. Although we are the first study to use a machine learning algorithm to construct a radiomics model, to predict the response of primary lesions of childhood HB to chemotherapy. However, radiomics is now being used to predict the prognosis of hepatoblasts (Jiang et al. 2021). Our results show that different models can predict the response of HB primary foci to chemotherapy, SVM model has the best prediction performance, with AUC and precision up to 0.830 and 0.774. The precision of the combined model combined with the clinical model can be up to 0.874 and 0.844, respectively. The combined model has better clinical applicability than the clinical model. It has been found, through the Delong test, that the combined model exhibits higher accuracy and better calibration in predicting preoperative chemotherapy response in HB, as compared to the clinical models.

**Fig. 6:**
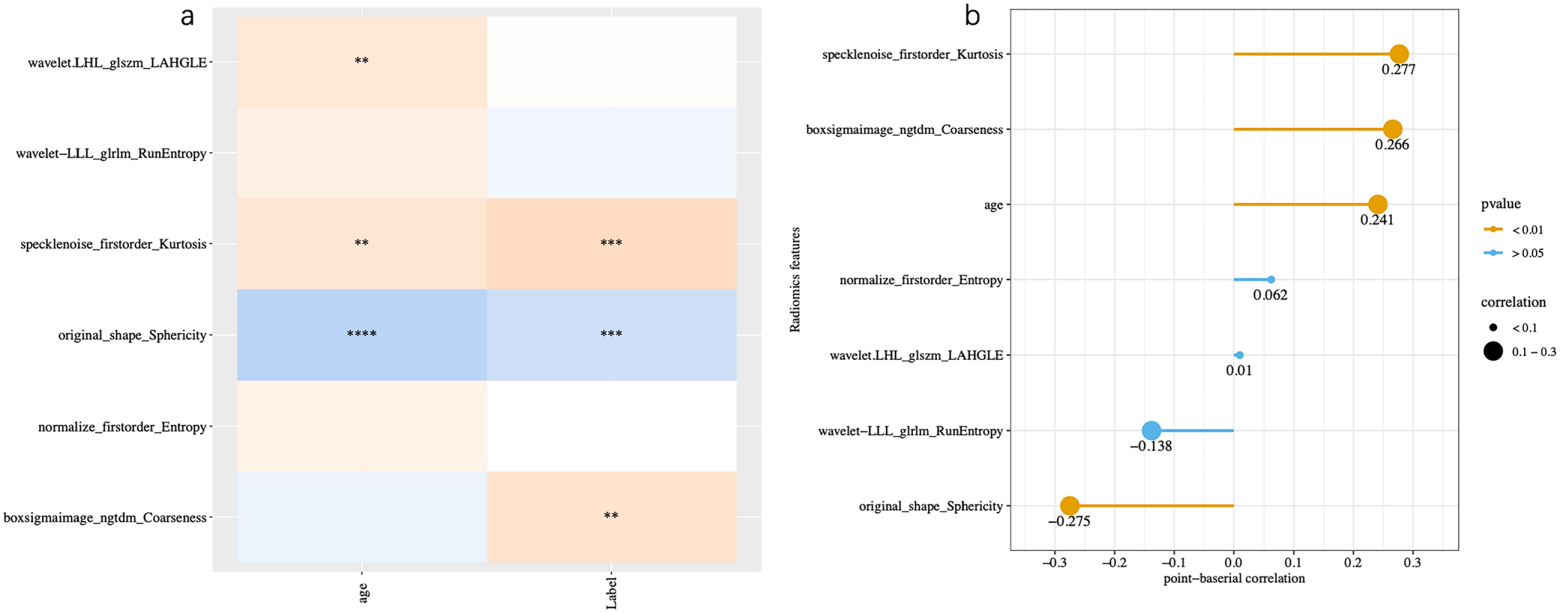
**a** Features selected, **b** selection of characterization coefficients and correlations

The study found that both radiomics shape features and texture features respond to the heterogeneity within the tumor tissue (Marusyk et al. 2012). The shape feature was used to describe the complexity of the identified tumors in the liver, to improving the sensitivity and specificity of the response prediction (Gillies et al. 2016; Gu et al. 2023), and the use of shape feature descriptions enhances the automatic prediction of tumors. The use of these features illustrates the complexity of the tumor and the tendency of the tumor to respond due to its rapid growth, complex shape, invasiveness, and high likelihood of non-responsiveness to preoperative chemotherapy. As per the research conducted by Olya Grove et al., it has been observed that a considerable amount of texture and shape features can be derived from the burrs of lung cancer. These features can effectively showcase the heterogeneity and complexity of the tumor and have been proven to be a strong predictor (Grove et al. 2015). In HB, the use of texture features improved the specificity and sensitivity of chemotherapy response prediction. A recent study (Chen et al. 2024) used unsupervised machine learning to cluster HBs based on their heterogeneity. The researchers found that HBs in different clusters differed in terms of clinical features and chemotherapy relevance. It is suggested that the heterogeneity of HBs’ images may correlate with clinical features and chemotherapy response. The likelihood of non-response to preoperative chemotherapy is high due to the rapid growth rate and complex morphology of the tumor, using these characteristics to illustrate the complexity of the tumor and the tendency to respond to the tumor. In our study, it was observed that first-order and second-order textural features (NGTDM and GLRLM) had a high correlation with chemotherapy response. This indicates that textural features can improve the model’s ability to detect tumor heterogeneity (Fig. 6).

Furthermore, the CECT image-based histologic analysis used in this study may provide valuable predictors.

Admittedly, this study has some shortcomings. First, as a retrospective study, this study included patients with inconsistent cycles of chemotherapy and was limited by its single-center retrospective design and small sample size, and it would be beneficial to consider expanding the sample size or conducting a multicenter study in the future. Second, it was recently discovered that the cytosolic fine transcriptome profile of HB tumors can predict drug sensitivity (Wu and Rangaswami 2022). Unfortunately, as this was a retrospective study, we only extracted raw radiomics features and did not explore the relationship between radiomics features and biogenetic information. In the future, studies with larger samples will help to further elucidate the predictive performance of screening-transformed radiomics features for response to neoadjuvant chemotherapy in HB. In summary, the CT-based radiomics model of the study was able to identify sensitivities before neoadjuvant chemotherapy for HB. This provides predictive value that may support clinical decision-making for patients with HB. In the future, it is important to test the model’s generalizability by externally validating it in test cohorts at different institutions using different device brands. This will demonstrate the model’s reproducibility and stability.

In conclusion, CECT-based imaging features and machine learning models can assess the response of HB primary foci to neoadjuvant chemotherapy, which can help in the development and decision-making of clinical treatments.

### Supplementary Information

Below is the link to the electronic supplementary material.

Supplementary file1 (XLSX 13 KB)

Supplementary file2 (XLSX 1937 KB)

Supplementary file3 (DOCX 17 KB)

## Data Availability

All data can be obtained from the supplementary files.
