# Supplementary material for: Predicting response of hepatoblastoma primary lesions to neoadjuvant chemotherapy through contrast-enhanced computed tomography radiomics
Source: J Cancer Res Clin Oncol. 2024 Apr 30;150(5):223. doi: 10.1007/s00432-024-05746-x (PMC11063102; doi:10.1007/s00432-024-05746-x)
Supplement: Supplementary file 3 — Supplementary file3 (DOCX 17 KB) [file 432_2024_5746_MOESM3_ESM.docx]

| **Supplementary Table S1**: 5-fold cross-validation for the best 5 group of best features. | | | | | |
| --- | --- | --- | --- | --- | --- |
| experimental group | fold 1 | fold 2 | fold 3 | fold 4 | fold 5 |
| Feaure name | specklenoise_firstorder_Kurtosis | specklenoise_firstorder_Kurtosis | specklenoise_firstorder_Kurtosis | discretegaussian_ngtdm_Strength | recursivegaussian_gldm_SmallDependenceLowGrayLevelEmphasis |
|  | original_shape_Elongation | boxsigmaimage_ngtdm_Coarseness | wavelet_ngtdm_wavelet.LLH.Busyness | boxsigmaimage_ngtdm_Busyness | binomialblurimage_glszm_LargeAreaEmphasis |
|  |  | laplaciansharpening_firstorder_MeanAbsoluteDeviation | wavelet_girlm_wavelet.LLL.RunEntropy | log_glszm_log.sigma.0.5.mm.3D.SizeZoneNonUniformityNormalized | wavelet_glrlm_wavelet.HHH.RunLengthNonUniformityNormalized |
|  |  | mean_glcm_ClusterShade | original_shape_Sphericity | wavelet_glszm_wavelet.LHL.LargeAreaHighGrayLevelEmphasis | original_glcm_ClusterProminence |
|  |  | boxmean_glszm_LargeAreaEmphasis | wavelet_firstorder_wavelet.LLH.Kurtosis | additivegaussiannoise_gldm_SmallDependenceLowGrayLevelEmphasis | wavelet_gldm_wavelet.HLL.DependenceEntropy |
|  |  | wavelet_glszm_wavelet.LHL.LargeAreaHighGrayLevelEmphasis | log_glrlm_log.sigma.4.0.mm.3D.RunEntropy | wavelet_firstorder_wavelet.LHH.Median | laplaciansharpening_firstorder_Skewness |
|  |  | original_firstorder_Median |  | curvatureflow_glrlm_ShortRunEmphasis | wavelet_glcm_wavelet.LLH.Imc1 |
|  |  | recursivegaussian_glszm_ZoneEntropy |  | specklenoise_firstorder_Kurtosis | original_shape_Maximum2DDiameterColumn |
|  |  | wavelet_glrlm_wavelet.LLL.RunEntropy |  | laplaciansharpening_glcm_ClusterShade | laplaciansharpening_firstorder_Uniformity |
|  |  | normalize_firstorder_Entropy |  | log_ngtdm_log.sigma.0.5.mm.3D.Strength | wavelet_glcm_wavelet.LLH.Imc1 |
|  |  |  |  | original_shape_Sphericity | wavelet_glszm_wavelet.LHL.LargeAreaHighGrayLevelEmphasis |
|  |  |  |  | normalize_firstorder_Entropy | normalize_firstorder_Entropy |
|  |  |  |  | boxsigmaimage_ngtdm_Coarseness | wavelet_glszm_wavelet.HHL.LargeAreaLowGrayLevelEmphasis |
|  |  |  |  | wavelet_firstorder_wavelet.LHH.Energy |  |
|  |  |  |  | specklenoise_ngtdm_Contrast |  |

| **Supplementary Table S2** Comparison of models in the training and validation cohorts. | | | |  |
| --- | --- | --- | --- | --- |
| Model | | Clinical model vs. Radiomics model | Clinical model vs. Combined model | Radiomics model vs. Combined model |
|  |  |  |  |  |
| p.training cohort | fold1 | 0.098 | 0.047 | 0.312 |
|  | fold2 | 0.076 | 0.009 | 0.116 |
|  | fold3 | 0.151 | 0.009 | 0.058 |
|  | fold4 | 0.039 | 0.027 | 0.648 |
|  | fold5 | 0.486 | 0.055 | 0.06 |
| p.validation cohort | fold1 | 0.882 | 0.277 | 0.148 |
|  | fold2 | 0.491 | 0.772 | 0.568 |
|  | fold3 | 0.537 | 0.76 | 0.366 |
|  | fold4 | 0.817 | 0.469 | 0.417 |
|  | fold5 | 0.019 | 0.035 | 0.262 |
| P values obtained using DeLong test | | | |  |
